# Therapeutic intervention of inflammatory/immune diseases by inhibition of the fractalkine (CX3CL1)-CX3CR1 pathway

**DOI:** 10.1186/s41232-016-0017-2

**Published:** 2016-06-15

**Authors:** Toshio Imai, Nobuyuki Yasuda

**Affiliations:** KAN Research Institute, Inc., 6-8-2 Minatojima-minamimachi Chuo-ku, Kobe, Hyogo 650-0047 Japan

**Keywords:** Fractalkine/CX3CL1, CX3CR1, Osteoclast, Rheumatoid arthritis, E6011

## Abstract

Inflammatory and immune responses are generated locally by the selective invasion and accumulation of the immune cells into the lesion site. The infiltration process of the immune cells into the tissue from the blood through the vascular endothelial cells is closely regulated by a number of chemotactic factors and cell adhesion molecules.

Fractalkine (FKN)/CX3CL1 is a membrane-bound chemokine possessing a chemokine/mucin hybrid structure and a transmembrane domain and has a dual function as an adhesion molecule and a chemoattractant. FKN is mainly expressed on activated endothelial cells, activated fibroblasts, and osteoblasts. Its receptor, CX3CR1, is expressed on cytotoxic effector lymphocytes, monocytes/macrophages, and osteoclasts. To date, a lot of key functional aspects of the FKN-CX3CR1 axis has been identified: (1) the rapid capture and firm adhesion of immune cells to vascular endothelial cells, (2) chemotaxis, (3) the enhancement of the transmigration to other chemokines, (4) the crawling behavior of the monocytes that patrol on vascular endothelial cells, (5) the retention of monocytes as the accessory cells of the inflamed endothelium to recruit inflammatory cells, and (6) the survival of the macrophage.

In this review, we will focus on the pathological role of FKN in rheumatoid arthritis (RA) and the physiological role of FKN on osteoclast differentiation. Furthermore, we will discuss the therapeutic potential of anti-FKN mAb for RA patients and its distinct mode of action from other cytokine inhibitors.

## Background

Rheumatoid arthritis (RA) is a long-lasting autoimmune disorder that primarily affects joints characterized by synovial hyperplasia and bone erosion associated with neovascularization, infiltration of pro-inflammatory cells, and increased cytokine production. Chemokines and their receptors control immune cell trafficking and are crucial for the inflammatory process. Fractalkine (FKN) is a unique membrane-bound chemokine possessing multiple biological functions. The FKN-CX3CR1 axis participates in the patrol for tissue damages and the quick mobilization and accumulation of immune cells to the sites of danger. The FKN-CX3CR1 axis is also involved in the pathogenesis in both bone-resorbing and inflammatory diseases. Taken together, FKN-CX3CR1 is expected to be a novel therapeutic target for RA by simultaneous direct inhibition of inflammation and bone resorption.

## Introduction

Rheumatoid arthritis (RA) is a chronic inflammatory disease characterized by the synovial hyperplasia, joint destruction, and massive infiltration of lymphocytes and macrophages into the synovium. Fibroblast-like synoviocytes (FLSs) also play a major role in the pathogenesis of RA by producing a variety of cytokines, chemokines, and matrix-degrading enzymes that mediate the interaction with neighboring inflammatory and endothelial cells. Chronic inflammatory environments are responsible for the progressive inflammation in the joints and the destruction of the articular cartilage and bone [1].

Chemokines are a family of small (8–10 kDa) proteins that play an important role in the recruitment and activation of immune cells. They are subdivided into four subfamilies, C, CC, CXC, and CX3C chemokines, based on the number and spacing of the amino-terminal, conserved cysteine residues. The biological effects of chemokines are mediated by their binding to the cognate receptors, seven-transmembrane G protein-coupled receptors (GPCRs). Over 50 chemokines and 19 receptors have been identified, for which complex ligand-receptor relationships are revealed with high redundancy [2].

Chemokines are originally identified as potent attractants for leukocytes such as neutrophils and monocytes and therefore are generally considered as mediators of acute inflammation (inflammatory chemokines). In addition, several chemokines have been found to be constitutively expressed in lymphoid and other tissues with individually characteristic patterns. Lymphocytes also express chemokine receptors with a cell-specific manner. Accumulating evidence indicates that chemokines are important not only in inflammation but also in the development, homeostasis, and functions of the immune system that should be shaped in a proper balance (immune or homeostatic chemokines).

In this review, we will discuss the roles of FKN, the only member of the CX3C chemokine family, on inflammatory/immune diseases and its potential as a new therapeutic target for RA.

## Functions of the FKN-CX3CR1 axis

FKN is a membrane-bound chemokine possessing a chemokine/mucin hybrid structure followed by a transmembrane domain [3]. This interesting structure allows FKN to work as an adhesion molecule in the membrane-bound form or as a chemoattractant in the soluble form shed by metalloproteases, a disintegrin and metalloproteinase domain-containing protein (ADAM) 10 or 17. Soluble FKN acts as a chemoattractant for monocytes, natural killer (NK) cells, and T cells. The membrane-bound FKN on endothelial cells mediates the rapid capture, integrin-independent firm adhesion, and activation of circulating leukocytes under flow by its direct binding to CX3CR1 [4, 5].

FKN is expressed on vascular endothelial cells and is strongly enhanced by the stimulation with pro-inflammatory cytokines, such as tumor necrosis factor-α (TNF-α), interleukin-1 (IL-1), and interferon-γ (IFN-γ). CX3CR1 is expressed on monocytes/macrophages and perforin+/granzyme B+ cytotoxic lymphocytes including NK cells and terminally differentiated cytotoxic T cells [6]. Soluble FKN preferentially induced the migration of cytotoxic effector lymphocytes, and the membrane-bound FKN promoted their subsequent migration to the secondary chemokines, such as the macrophage inflammatory protein-1β/CCL4 or IL-8/CXCL8. Thus, FKN expressed on the inflamed endothelium functions as a vascular regulator for cytotoxic effector lymphocytes. Interestingly, it is revealed that a subset of monocytes patrols healthy tissues through long-range crawling on the resting endothelium by the intravital imaging of blood monocytes [7]. This unique behavior depends on the physical interaction-mediated integrin LFA-1 and CX3CR1 and is required for rapid accumulation of the monocytes to the site of danger to initiate early immune response. Nr4a1-dependent Ly6C^low^ CX3CR1^high^ monocytes scan capillaries and scavenge micrometric particles from their luminal side in a steady state in the kidney cortex. Importantly, a local TLR7-dependent danger signal increases the retention time of Ly6C^low^ CX3CR1^high^ monocytes on the endothelium. These tethered monocytes are then activated and function as “accessory cells” of the endothelium by orchestrating the focal necrosis of endothelial cells by recruiting neutrophils and the in situ phagocytosis of cellular debris by monocytes [8]. The FKN-CX3CR1 axis also has been reported to increase the survival of microglia and smooth muscle cells by Akt activation in a PI3K-dependent manner [9]. Lionakis et al. demonstrated that CX3CR1 promotes resident macrophage survival by inhibiting caspase-dependent apoptosis in the kidney in a mouse model of systemic candidiasis [10].

**Fig. 1:**
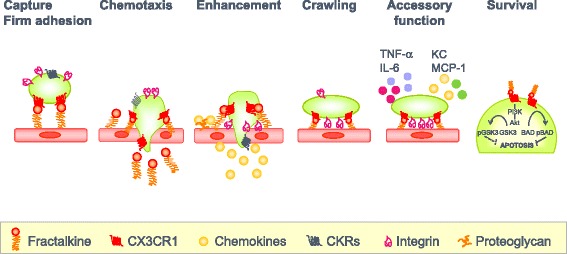
Multiple functions of FKN. Many key functional aspects of the FKN-CX3CR1 axis have been identified: (1) the rapid capture and firm adhesion of immune cells to vascular endothelial cells, (2) chemotaxis, (3) the enhancement of the transmigration to other chemokines, (4) the crawling behavior of the monocytes that patrol on vascular endothelial cells, (5) the retention of monocytes as the accessory cells of the inflamed endothelium to recruit inflammatory cells, and (6) the survival of the macrophage

These results indicate that the FKN-CX3CR1 axis participates in the patrol for tissue damages in normal conditions and the quick mobilization and accumulation of effector cells to the sites of danger (Fig. 1).

## Role of the FKN-CX3CR1 axis in the pathogenesis of RA

FKN is expressed on fibroblast-like synoviocyte (FLS) cells and endothelial cells in the RA synovium and contributes to the accumulation of T cells and macrophages, which express CX3CR1. The interaction between FKN and CX3CR1 is involved in the adhesion of the inflammatory cells to endothelial cells, their migration into the synovium, and cytokine production [1]. Nanki et al. showed that the peripheral blood CX3CR1-expressing CD4+ and CD8+ T cells preferentially produce IFN-γ, TNF-α, granzyme A, and perforin and that these cells are increased in patients with RA [11]. Furthermore, FKN expression is up-regulated in endothelial cells and FLSs in the synovium of RA patients, but not in the osteoarthritis synovium. Thus, the FKN/CX3CR1 axis is likely to play an important role in the preferential infiltration of Th1 and Tc1 cells into the RA synovium, which contributes to the pathogenesis of RA.

Macrophages are the primary source of pro-inflammatory cytokines. A high percentage of macrophages within the rheumatoid synovium express the CX3CR1 receptor. Circulating CD16+ monocytes express higher levels of CX3CR1 than CD16− monocytes in both RA patients and healthy subjects. High levels of CX3CR1 expression are also seen in CD16+ monocytes localized to the lining layer in RA synovial tissue, and soluble FKN efficiently induced the chemotaxis of these cell populations [12, 13]. Yano et al. postulated that the recruitment of CD16+ monocytes may be the result of the chemoattractant properties of FKN. Soluble FKN also induces IL-1 and IL-6 secretion from activated monocytes suggesting a crucial pro-inflammatory effect of FKN on monocyte function [12].

FLSs, which are resident cells in the sublining region and markedly expanded in the RA synovium, have been linked with a number of deleterious effects in RA. FLSs produce pro-inflammatory cytokines, exhibit a capacity for antigen presentation, and induce T cell expansion [14]. FKN is also expressed on cultured synovial fibroblasts and hyperplastic synoviocytes in RA. The senescent CD4+CD28− T cells that accumulate in the RA synovium aberrantly express CX3CR1. FKN, which is induced on FLSs by stimulation with pro-inflammatory cytokines, strongly induces the adhesion of CD4+CD28−CX3CR1+ T cells, provides survival signals and amplified proliferation, and stimulates the production of pro-inflammatory cytokines as well as the expulsion of cytoplasmic granules [15]. Thus, membrane-bound FKN may act as a co-stimulatory signal for CD4+CX3CR1+ T cells in the RA synovium.

In the collagen-induced arthritis (CIA) model in mice, the prophylactic treatment of anti-FKN monoclonal antibody (mAb) significantly improves the clinical arthritis score and reduces the infiltration of inflammatory cells, and bone erosion in the synovium, suggesting that anti-FKN mAb ameliorates arthritis by inhibiting the infiltration of inflammatory cells into the synovium [16]. In addition, our recent studies have shown that the therapeutic treatment of anti-FKN mAb also meliorates arthritis symptoms and radiological score in the CIA model (manuscript in preparation).

## Role of the FKN-CX3CR1 axis in bone destruction

Osteoclast precursors selectively express CX3CR1, whereas FKN is expressed on osteoblasts. FKN on the osteoblasts is involved in the osteoblast-induced osteoclast differentiation [17]. Soluble FKN induces the migration of bone marrow cells containing osteoclast precursors, whereas immobilized FKN mediates the firm adhesion of osteoclast precursors. Furthermore, the blockade of FKN efficiently inhibits osteoclast differentiation from mouse bone marrow cells when co-cultured with osteoblasts. Consistently, an in vivo experiment in neonatal mice shows that anti-FKN mAb significantly suppresses bone resorption by reducing the number of bone-resorbing mature osteoclasts.

In the analysis of the femoral bone tissues of heterozygous CX3CR1−EGFP knock-in mice, CX3CR1−EGFP+ cells are shown to differentiate into tartrate-resistant acid phosphatase (TRAP)+ mature osteoclasts. CX3CR1−EGFP+ but not CX3CR1−EGFP− cells sorted from bone marrow cells efficiently differentiate into TRAP+ mature osteoclast-like cells in vitro in the presence of RANKL, indicating that CX3CR1+ cells in the bone marrow are osteoclast precursors [18, 19].

The role of the FKN-CX3CR1 axis in osteoclast recruitment and osteoclastogenesis is evaluated by an irradiated murine model. FKN is dramatically up-regulated in the skeletal vascular endothelium after ionizing radiation (IR). The induced FKN promotes the recruitment of circulating CX3CR1+ osteoclast precursors toward the bone remodeling surface in the irradiated bones and enhances subsequent bone resorption. In vivo experiments also show that the blockade of the FKN-CX3CR1 axis ameliorates osteoclastogenesis and prevented bone loss after IR [20].

Collectively, FKN plays an important role in osteoclast recruitment and differentiation, possibly through its dual functions as a chemotactic factor and an adhesion molecule for CX3CR1+ osteoclast precursors. The FKN-CX3CR1 axis may be a novel target for the therapeutic intervention of bone-resorbing diseases such as RA and osteoporosis.

## Development of the first humanized anti-FKN mAb, KANAb001 (E6011)

KANAb001 (E6011) is the first humanized anti-FKN mAb, generated by KAN Research Institute, Inc. Currently, phase 1/2 clinical studies of E6011 are ongoing in both RA and Crohn’s disease in Japan by Eisai Co., Ltd. Recently, we have reported that E6011 is safe and well tolerated and has a promising efficacy in active RA patients with MTX or TNF inhibitor-inadequate response (MTX-IR or TNFi-IR) at the American College of Rheumatology 2015. While further clinical studies are required, the results obtained to date indicate that a novel biological DMARD targeting the FKN-CX3CR1 axis will be clinically beneficial for active RA patients.

**Fig. 2:**
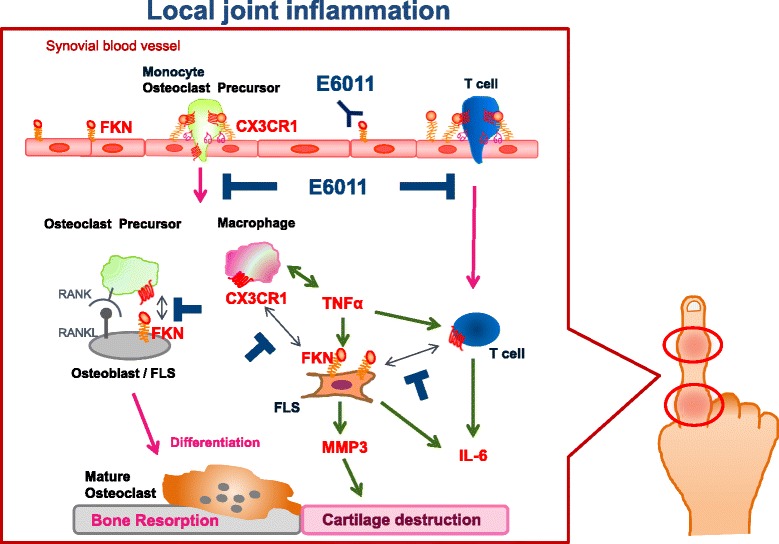
E6011 targets CX3CR1+ cells in the inflammatory sites in RA. The interaction of the osteoclast precursor with osteoblasts via FKN-CX3CR1 promotes the osteoclast differentiation. The macrophage may be recruited by FLS-induced chemokine production through FKN-CX3CR1. In turn, the macrophage helps to activate the synovial sublining fibroblasts through the production of inflammatory cytokines such as TNF-α. CX3CR1 expressed on the cytotoxic effector CD4+ T cell binds to FKN on the fibroblast. And then, T cell-FLS communication activates the TNF-α production. TNF-α up-regulates FKN production as a growth factor of synovial fibroblasts, and TNF-α also induces the MMP3 expression, matrix metalloproteinase. Taken together, the interaction of FLS with the macrophage and T cells through FKN-CX3CR1 may contribute to the enhancement of inflammation and joint destruction

Most of current marketed mAbs inhibit specific cytokines and cytokine receptors, and JAK inhibitors block the effect of multiple cytokines by targeting cytokine signaling. On the other hand, E6011 targets the cell trafficking of immune cells, which produce multiple pro-inflammatory cytokines in the local inflamed sites. Previous studies have demonstrated that anti-FKN mAb inhibit the migration of both CX3CR1+ macrophages producing pro-inflammatory cytokines (TNF-α, GM-CSF, and IL-6) and CX3CR1+ cytotoxic effector T cells containing cytotoxic molecules (granzyme B and perforin). These results indicate that anti-FKN mAb has a potential to block the most upstream step of the inflammation cascade in the local inflamed region. In addition, anti-FKN mAb suppresses the bone resorption by inhibiting osteoclast differentiation. Taken together, E6011 is expected to have strong preventive effects on joint destruction with a unique dual mode of action based on the inhibition of the immune cell trafficking and the direct suppression of osteoclastogenesis in local environments (Fig. 2).

## Conclusions

FKN is a unique chemokine possessing a dual function as a chemoattractant and an adhesion molecule for CX3CR1-expressing monocytes, cytotoxic effector lymphocytes, and osteoclast precursors. Increasing evidence indicates that FKN is involved in the pathological roles of inflammatory disease such as RA. Now, clinical trials of E6011, the first humanized anti-FKN mAb, are ongoing in Japan. It is expected that E6011 will open up the possibility of a new therapeutic strategy for the treatment of RA with a novel mode of action distinct from other cytokine/cytokine receptor inhibitors (infliximab, tocilizumab, etc.) and modulator of T cell co-stimulation (abatacept).

## Abbreviations

ADAM: metalloproteinase domain-containing protein
FKN: fractalkine (FKN)
FLSs: fibroblast-like synoviocytes
IFN-γ: interferon-γ
IL-1: interleukin-1
IR: ionizing radiation
mAb: monoclonal antibody
MTX: methotrexate
NK cells: natural killer cells
RA: rheumatoid arthritis
TNF-α: tumor necrosis factor-α
TRAP: tartrate-resistant acid phosphatase
